# Poly(lactic-co-glycolic acid) microsphere production based on quality by design: a review

**DOI:** 10.1080/10717544.2021.1943056

**Published:** 2021-06-28

**Authors:** Yabing Hua, Yuhuai Su, Hui Zhang, Nan Liu, Zengming Wang, Xiang Gao, Jing Gao, Aiping Zheng

**Affiliations:** State Key Laboratory of Toxicology and Medical Countermeasures, Beijing Institute of Pharmacology and Toxicology, Beijing, China

**Keywords:** Poly(lactic-co-glycolic acid), sustained-release, continuous manufacturing, quality by design, critical quality attributes, microsphere

## Abstract

Poly(lactic-co-glycolic acid) (PLGA) has garnered increasing attention as a candidate drug delivery polymer owing to its favorable properties, including its excellent biocompatibility, biodegradability, non-toxicity, non-immunogenicity, and mechanical strength. PLAG are specifically used as microspheres for the sustained/controlled and targeted delivery of hydrophilic or hydrophobic drugs, as well as biological therapeutic macromolecules, including peptide and protein drugs. PLGAs with different molecular weights, lactic acid (LA)/glycolic acid (GA) ratios, and end groups exhibit unique release characteristics, which is beneficial for obtaining diverse therapeutic effects. This review aims to analyze the composition of PLGA microspheres, and understand the manufacturing process involved in their production, from a quality by design perspective. Additionally, the key factors affecting PLGA microsphere development are explored as well as the principles involved in the synthesis and degradation of PLGA and its interaction with active drugs. Further, the effects elicited by microcosmic conditions on PLGA macroscopic properties, are analyzed. These conditions include variations in the organic phase (organic solvent, PLGA, and drug concentration), continuous phase (emulsifying ability), emulsifying stage (organic phase and continuous phase interaction, homogenization parameters), and solidification process (relationship between solvent volatilization rate and curing conditions). The challenges in achieving consistency between batches during manufacturing are addressed, and continuous production is discussed as a potential solution. Finally, potential critical quality attributes are introduced, which may facilitate the optimization of process parameters.

## 1. Introduction

Currently, various biodegradable materials, including natural and synthetic polymers, have been investigated as candidates for drug delivery (Hossain et al., 2015). Natural polymer materials are currently the primary sources used in the preparation of microspheres; however, their disadvantages and limitations, including high immunogenicity and the presence of impurities, must be overcome before their clinical application in drug delivery. Meanwhile, synthesized biodegradable polymers have become alternatives to the natural polymers. Indeed, several synthetic and semisynthetic biodegradable polymers have recently been developed, including methylcellulose (MC), ethyl cellulose (EC), carboxymethyl cellulose (CMC), and cellulose acetate phthalate (CAP). Their versatility, biocompatibility, and tunable biodegradation rate allow synthetic biodegradable polymers to be readily formulated into various drug carrying systems. Among the synthetic biodegradable polymers, poly(lactic-co-glycolic acid) (PLGA) has generated great interest due to its excellent biocompatibility, biodegradability, non-toxicity, non-immunogenicity, and mechanical strength. As such, PLGA has been approved by the Food and Drug Administration (FDA), as well as the European Medicines Agency (EMA), for orthopedics fixation, medical surgical sutures, and parenteral sustained-release drug delivery systems (Biondi et al., 2008; Snejdrova et al., 2020). Since its discovery, PLGA has found varied applications in the field of medicine (Astete & Sabliov, 2006). Over the past few decades, PLGA microspheres have become among the most successful complex parenteral drug formulations on the market. In fact, the FDA has approved over 10 PLGA-mediated sustained-release microsphere formulations, including the peptide-containing microspheres, Lupron Depot^®^ (leuprolide), Sandostatin^®^ LAR (octreotide acetate), Nutropin^®^ (depot somatropin), and Trelstar™ Depot (triptorelin); as well as the small molecule-containing microspheres Risperdal Consta^®^ (risperidone) and Vivitrol^®^ (naltrexone) (Allen & Evans, 2020). These depot formulations provide sustained drug release over a period of a few weeks to months. Despite the tremendous efforts invested into formulating drug-loaded PLGA microspheres, few have been developed and approved for clinical use (Wang et al., 2016), which may be due to our insufficient understanding of the polymer and PLGA microsphere manufacturing processes.

Quality by design (QbD) is ‘a systematic approach to development that begins with pre-defined objectives and emphasizes product and process understanding and process control, based on sound science and quality risk management’ (Zhang et al., 2020). That is, it requires a sound understanding of the product and process to assure a high quality final product. This design process involves the construction of models to correlate input parameters with output. The design space is defined by the mathematical relationship between the critical process parameters (CPPs) and critical material attributes (CMAs) and critical quality attributes (CQAs). Therefore, the manufacturing process is well understood, allowing the final product to meet the quality target product profile (QTPP) (Politis et al., 2017). The CQAs of PLGA microspheres (drug loading, particle size, glass transition temperature (Tg), and porosity) are sensitive to minor manufacturing changes, which can influence the drug release characteristics (Andhariya et al., 2017). Thus, the properties of microspheres must be characterized to ensure consistency in the formulation process and performance.

The critical properties of PLGA, such as lactic acid/glycolic acid (LA/GA) ratio, molecular weight (Mw) distribution, and end-group, can influence the rate, and mechanism, of drug release from the microspheres. Similarly, the method of PLGA synthesis, as well as the catalyst used, can affect product performance. It is, therefore, vital to understand the CPPs and CMAs for PLGA to facilitate production of microspheres with high reproducibility. Moreover, during the manufacturing process, the PLGA polymer can degrade, resulting in changes in the formulated product and failure in the equivalence test. Indeed, several studies have reported key quality properties desired in PLGA microspheres; however, few new PLGA microspheres have been approved for clinical use. This may be due, in part, to our poor understanding of PLGA and its degradation process. However, it may also be attributed to the intricate formulation process, which may impede development of PLGA-based drug products, making it highly challenging to obtain regulatory approval. The complexity of the manufacturing process not only has the potential to increase the risk of altering active drug properties but can also affect the microsphere product performance. Indeed, microspheres of the same composition, produced in separate small batches can have different quality and release properties. Therefore, it is important to understand the details of the manufacturing process that can affect the properties of PLGA microspheres.

Herein, we investigate the example of the traditional emulsion-solvent evaporation technology to analyze the prescription and process of PLGA microsphere production. We also describe the synthesis and degradation principles of PLGA, as well as the interaction between PLGA and active drugs. The effects of microcosmic conditions on macroscopic properties (drug loading, entrapment efficiency, particle size, Tg, porosity, and morphology of PLGA microspheres) are also analyzed in detail, including the organic phase (organic solvent, PLGA, and drug concentration), continuous phase (emulsifying ability), emulsifying stage (organic phase and continuous phase interaction, homogenization parameters), and solidification process (the relationship between solvent volatilization rate and curing conditions). Subsequently, we propose that continuous preparation and production of PLGA microspheres as a potential solution to the current challenges facing their manufacture. Finally, we propose CQAs that we believe are important in the production of PLGA microspheres.

## 2. PLGA hydrolysis and drug release from microspheres

**Figure 1.**
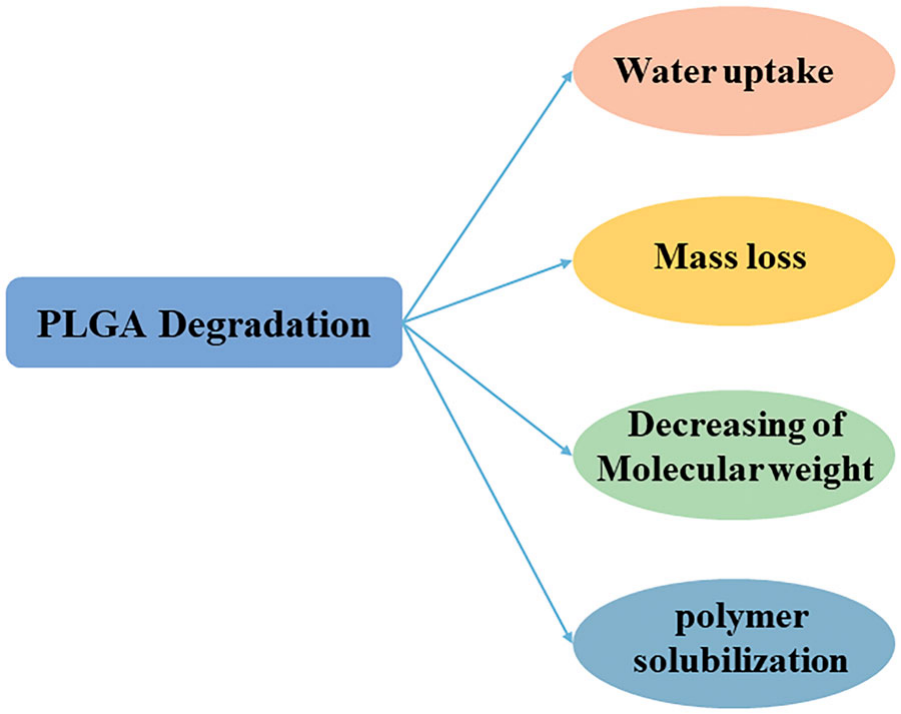
The mechanism of PLGA degradation.

In an aqueous environment, the ester linkages in PLGA are hydrolyzed, and the polymer undergoes water uptake, mass loss, decreased Mw and bulk, or heterogeneous erosion (Figure 1). This occurs in four major stages, namely, the hydration stage, initial degradation, constant degradation, and polymer solubilization. In the hydration stage, degradation is initiated by water uptake by the polymer. Hydrogen bonding and Van der Waals forces destroy the primary and secondary structures of PLGA, leading to production of acidic oligomers (A & B, 2001) and a decrease in the Tg. The initial degradation phase then occurs, wherein covalent bonds in the polymer backbone rupture to form oligomers with acidic end groups, resulting in the loss of mechanical strength and a decrease in Mw. Thereafter, mass and integrity of the polymer are lost through diffusion of acidic oligomers in the constant degradation phase, which results in accelerated water absorption. The final stage is polymer solubilization, during which oligomers are cleaved to form water-soluble molecules (Gentile et al., 2014).

**Figure 2.**
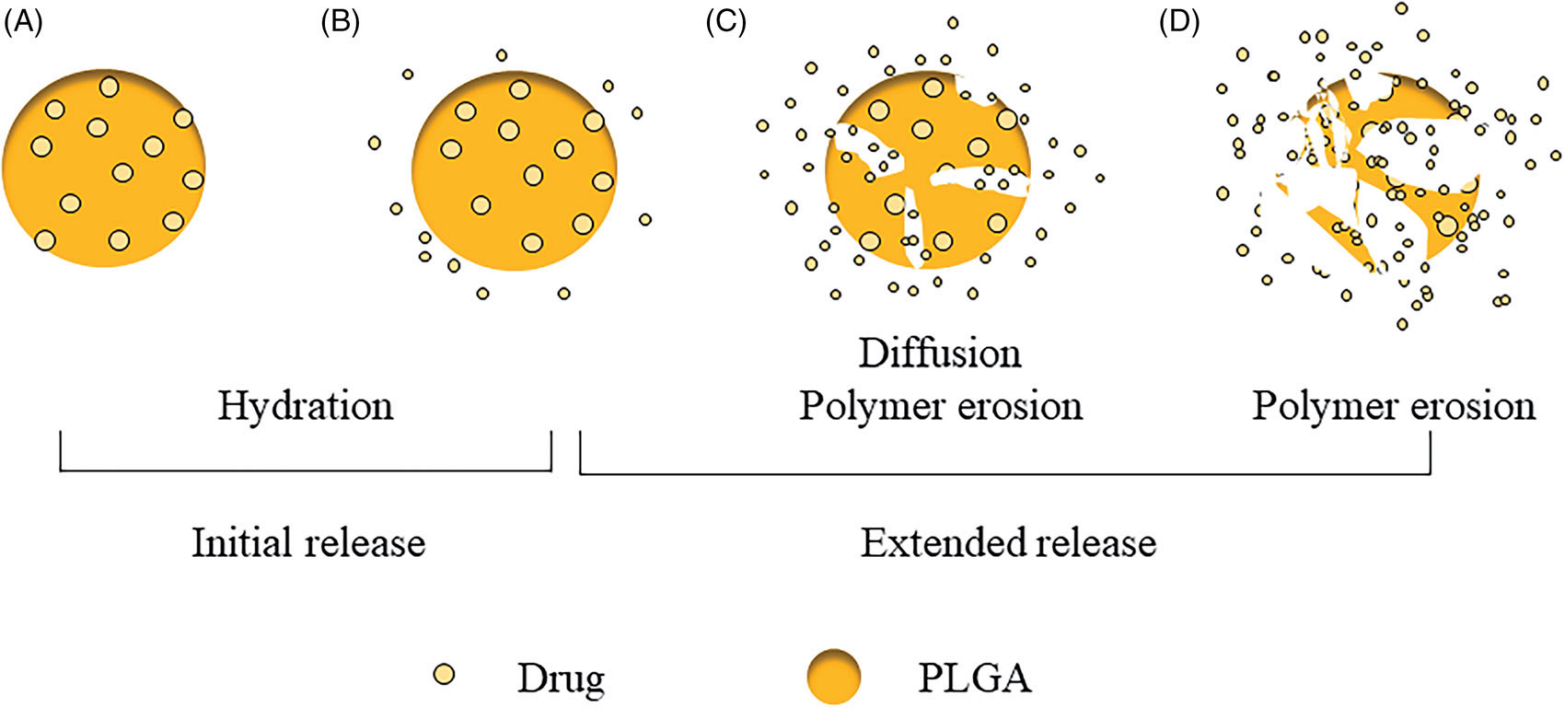
Degradation mechanism of PLGA microspheres.

In an aqueous environment, PLGA microspheres exhibit drug release in three release phases (Figure 2). The diffusion of biological fluids into PLGA microparticles is much more rapid than the subsequent ester hydrolysis, and PLGA degradation occurs via exposure to aqueous media (Siepmann et al., 2005). Initial release occurs when the microsphere encounters the aqueous medium and becomes wet, allowing the drug molecules on, or near, the surface of the microsphere to dissolve and be released into the medium. Additionally, water from the medium diffuses into the microsphere (swelling) owing to the increased osmotic pressure. Interestingly, the initial microsphere swelling can, reportedly, induce the formation of a ‘skin’ layer on the surface due to pore-closing, thereby delaying initial drug release and effectively causing an apparent lag phase (Wang et al., 2002). The next stage is the hydration phase in which the acidic environment, formed by accumulation of oligomeric acids in the microspheres, leads to autocatalysis; microspheres often begin degrading from the ‘inside,’ indicating the presence of a pH gradient from the interior (low pH) to the exterior surface (high pH) of the microspheres (Fu et al., 2000; Zolnik & Burgess, 2007; Liu et al., 2012). In this phase, the microspheres continue to hydrate and experience a steady decrease in Mw of the polymer. Moreover, the burst release, swelling, and water uptake in clonidine loaded PLGA microspheres have been reported to occur during the release phase owing to initial microsphere swelling (Messaritaki et al., 2005; Gaignaux et al., 2013; Gasmi et al., 2015a,b). Microsphere swelling may result from polymer chain relaxation, caused by the increased osmotic pressure that results from accumulation of dissolved drug and degradation species (Brunner et al., 1999). The high porosity of the formulation makes it easy for water to access the ester linkage of the polymers, and for drugs to escape the microspheres, which may be a primary cause of the shorter lag phase observed for this formulation compared to those that are less porous. The hydration phase is followed by the continuous release phase, in which the encapsulated drug diffuses out of the degraded polymer microsphere. This is controlled by polymer erosion until the drug is completely released. In this phase, most of the unreleased drug molecules are directly exposed to the medium; thus, drug release is likely to be accelerated (Kumar & Palmieri, 2010).

In brief, the diffusion of biological fluids into PLGA microparticles is significantly more rapid than the subsequent ester hydrolysis, while PLGA degradation occurs via exposure to aqueous media (‘bulk erosion’). Due to concentration gradients, the generated acids subsequently diffuse out of the microparticles into the surrounding bulk fluid, where they are neutralized. Meanwhile, bases, from the surrounding environment, diffuse into the system where they neutralize the generated acids. However, diffusional processes are relatively slow and, depending on the length of the diffusion pathways and mobility of the involved species, the rate at which the acids are generated can be higher than the rate at which they are neutralized. Consequently, the micro-pH within the system can become significantly decreased.

## 3. A comprehensive characterization of PLGA

The physical and chemical properties of PLGA microspheres are characterized by the CQA. Most of the difficulties faced in the development of PLGA sustained-release microspheres stem from the lack of understanding of PLGA at the molecular level. Thus, comprehensive characterization of PLGA is required to aid the development of polymer-based products.

### 3.1. PLGA synthesis

**Figure 3.**
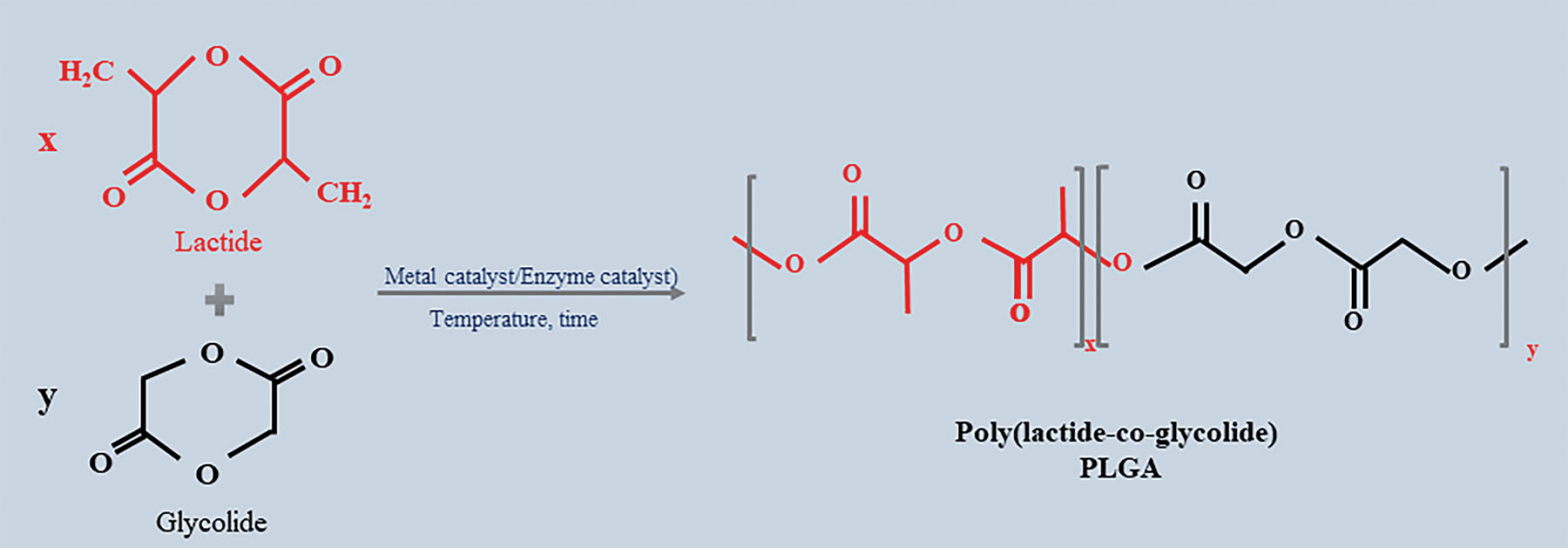
Chemical structure of poly(lactic-co-glycolic acid) and its monomers, and the synthesis of PLGA.

PLGA can be synthesized from its monomers LA and GA, at various ratios (Figure 3). Polymerization can be achieved in two distinct ways: (1) direct polycondensation of LA and GA and (2) ring-opening polymerization (ROP) of the cyclic dimers, lactide, and glycolide.

Synthesis by ROP is more difficult; however, this method allows for controlled polymerization and can produce high Mw polymers with well-defined chemical, structural, thermal, and mechanical properties (Erbetta et al., 2012; Raquez et al., 2006). Lactides and glycolides are cyclic dimers obtained via dehydration of LA and GA. LA is a methyl-substituted GA or 2-hydroxypropanoic acid that can be produced in d and l forms. GA is 2-hydroxyethanoic acid (Kapoor et al., 2015).

To enhance polymerization kinetics, it is necessary to select a suitable catalyst capable of producing a high yield and suitable reaction rate. Currently, only stannous (II) chloride and stannous (II) 2-ethylhexanoate (Sn (Oct)_2_) have been approved by the FDA for the catalysis of ROP. Sn (Oct)_2_ effectively catalyzes ROP by reactive extrusion and is generally combined with proton compounds to promote its compatibility with the monomer medium, while also preventing evaporation. The final Mw of polyester is then determined after synthesis. During the polymerization of PLGA, successive monomeric units are linked together by ester linkages, thus yielding a linear, aliphatic polyester. PLGA can be synthesized as either random or block copolymers, thereby imparting additional polymer properties.

### 3.2. PLGA properties

**Figure 4.**
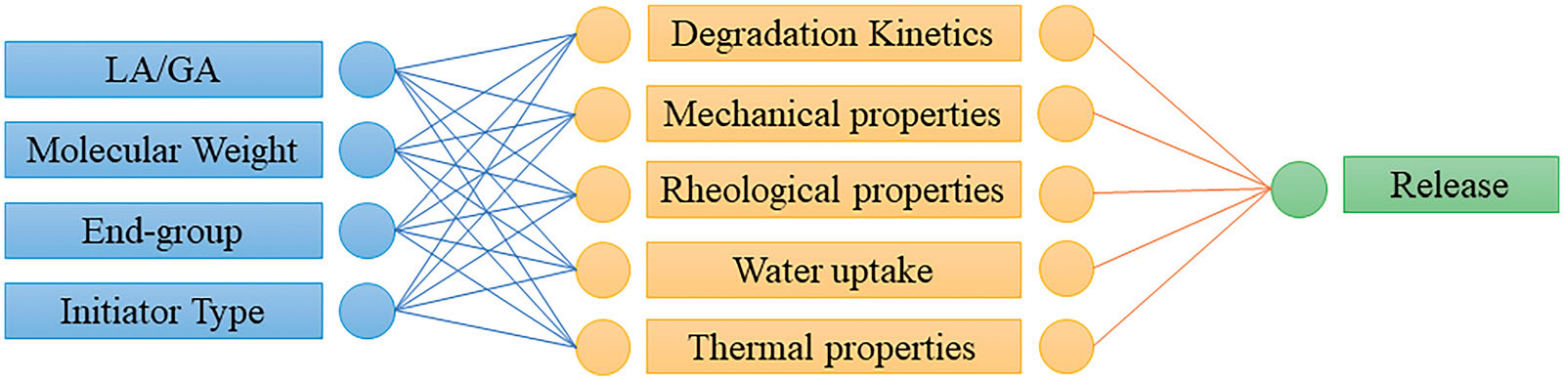
Adjusting the polymer to control the performance.

Modification of the LA/GA ratio, Mw, terminal group, and synthetic parameters of PLGA facilitates the engineering of precise polymer properties, such as degradation kinetics, mechanical properties, rheological properties, thermal properties, and water uptake and release profiles (Figure 4).

Generally, a higher percentage of lactide units produces a polymer that degrades slower in an aqueous medium (Lamprecht et al., 2000). Meanwhile, a higher proportion of glycolide units produces a more hydrophilic polymer, with a higher rate of degradation. PLGA may have either esters or acids as end groups; ester end groups make the polymer more resistant to hydrolytic degradation. The Mw of PLGA also affects degradation and the drug release properties (Andhariya et al., 2016). That is, higher Mw polymers exhibit greater mechanical strength and undergo slower degradation. Furthermore, differences in the synthesis and purification methods used by different manufacturers bring about variation in the monomer sequence and in the type and quantity of residual solvent.

#### 3.2.1. Monomer composition: the GA/LA ratio

PLGA is composed of a GA and LA chain linked by ester bonds. GA is the smallest α-hydroxy acid and, thus, is highly soluble in water. Therefore, an ester linkage containing GA (GA–GA or GA–LA) is more hydrophobic, resulting in higher priority cleavage than that of LA–LA linked esters. Additionally, the presence of methyl side chains in LA makes it more hydrophobic than GA, thus, lactide-rich PLGA copolymers are less hydrophilic, absorb less water, and subsequently degrade more slowly (Schliecker et al., 2003). We found that PLGA has a high rate of degradation when the ratio of LA/GA is 50/50 and the rate decreases as the proportion of LA is increased. Furthermore, the degradation of GA produces more acidic oligomers, which exhibit a higher rate of monomer cracking. The hydrolyzed product has a high local concentration of hydrolysates in the structure, which autocatalyze internal degradation and polymer erosion (Fu et al., 2000; Zolnik & Burgess, 2007; Schädlich et al., 2014). It is, therefore, vital to select the appropriate LA/GA ratio to achieve the desired *in vivo* drug release kinetics.

Modulation of the polymer composition can also affect other properties that impact release kinetics. For instance, a lower proportion of lactide than galactide in the copolymer may decrease the Tg (Graham et al., 1999), which is an indicator of the chain structure rigidity and is generally above 37 °C. Hence, careful regulation of the monomer composition is crucial (Passerini & Craig, 2001; Wu et al., 2006).

#### 3.2.2. The terminal group

A PLGA chain with a relatively low averaged Mw of 2 kg/mol consists of approximately 30 repeat units, approximately 7% of which form terminal groups, and 93% are reactive (Machatschek & Lendlein, 2020; Siepmann et al., 2005). The PLGA terminal groups influence the degradation rate. For instance, carboxyl terminal groups can catalyze the hydrolysis of ester bonds, thus producing more acidic groups and establishing an autocatalytic cycle that accelerates polymer degradation. Therefore, the rate of PGLA degradation with carboxyl terminal groups is higher than that with ester terminal groups (Lanao et al., 2011). Furthermore, the end groups significantly affect drug encapsulation efficiency and loading capacity of the polymer. Wang et al. studied the water contact angle of the polymer to find that PLGA with ester terminal groups is more hydrophobic. This allows it to encapsulate increased quantities of drugs, possibly as a result of the delayed hydrolysis of the PLGA microspheres during the curing process. Moreover, they reported that the effect of PLGA end groups on low Mw polymers is more pronounced. At a given mass, PLGA with lower Mw is likely to contain more acidic groups than higher Mw PLGA due to difference in the densities of carboxyl terminal groups for PLGAs of different Mw (Wang et al., 2019). In cases where terminal groups have an autocatalytic effect, the initial Mw distribution can influence PLGA degradation, with polydisperse polymers having more terminal groups than monodisperse polymers.

#### 3.2.3. Molecular weight

The Mw of PLGA is a key attribute that affects particle size, drug loading, initial release, and release duration of microspheres. To ensure high encapsulation efficiency and good sustained-release properties, selection of the appropriate PGLA Mw range is required. Considering that PLGA manufacturers are not required to provide the sequence information or Mw distribution of the polymer, however, must disclose the Mw (expressed in terms of viscosity of the polymer), the Mw distribution can be determined using gel permeation chromatography (GPC), multi-angle light scattering (MALS), intrinsic viscosity, or osmotic pressure. Of these platforms, only the GPC method can directly detect Mw distribution, making it the most commonly employed method (Rawat & Burgess, 2011). The Mw of PLGA in microspheres may be affected by the manufacturing process. For instance, Reich found that the Mw of PLGA in microspheres decreased significantly after homogenization or ultrasonic mixing (Reich, 1998). Additionally, Cha & Pitt formulated meperidine-loaded PLGA microspheres and found that the drug catalyzed the hydrolytic cleavage of PGLA in the manufacturing process, causing a decrease in the Mw and a faster rate of drug release (Cha & Pitt, 1989). Selmin et al. (2012) and Zolnik et al. (2006) formulated risperidone microspheres using PLGA of different Mw. They reported that the microsphere sizes varied when drug loading was kept constant and the Mw of PLGA decreased by varying extents after it was converted into microspheres.

The drug release mechanism is also reportedly influenced by the polymer Mw. That is, drug release from low Mw PLGA microspheres is generally diffusion-controlled, whereas release from high Mw PLGA microspheres is often governed by polymer erosion, along with drug diffusion (Zolnik et al., 2006).

### 3.3. Polymer chain branching and the use of initiators in synthesis

PLGA chains may be linear or branched. These differences in the structure can affect the degradability of the polymer and, in effect, the sustained-release effect of the formulation. Branched PLGAs are particularly well-suited as drug carriers owing to their short degradation time (few hours or days).

Linear PLGA is formed by ROP. Branched PLGAs can take several forms, including star or dobby shapes. ROP can be modified to synthesize star-shaped PLGA, by adding an initiator containing polyhydroxy groups (e.g. glucose) as the core and activating the hydroxyl groups. Indeed, glucose-initiated PLGA (Glu-PLGA) has been used in Sandostatin^®^ LAR Depot (octreotide acetate injectable suspension) approved by the U.S. FDA in 1998 (Hadar et al., 2019).

The molar mass, branching ratio, and inherent viscosity of the polyesters can be determined using the triple method: size exclusion chromatography (Podzimek, 2014), MALS photometry (Podzimek, 2013), and on-line viscometry (Martiska et al., 2019). The branching of the polymer chain can be described by the branching ratio *g*′ (Zimm & Stockmayer, 1949), which is obtained by dividing the mean square radius of the branched molecule by the ratio of the mean square radius of the linear molecule and its molar mass. The required root mean square (RMS) radius, is the radius of gyration, obtained from MALS and molar mass.

The branching ratio *g*′ is the ratio of intrinsic viscosity of the branched molecule and the linear molecule of the same molar mass (Zimm & Kilb, 1996). The *g*′ value is applied for linear polymers and decreases with branching. An alternative size parameter must be used in the case of smaller molecules, as the RMS radius cannot be reliably determined for radii smaller than 10 nm. *g*′ can be calculated as follows:

(1) $$g^{'}={\left(\frac{3f-2}{f^{2}}\right)}^{0.58}\frac{0.724-0.015(f-1)}{0.724}$$

where *f* represents the number of random length arms in star polymers and *f* can be directly calculated from *g*′. The limitation here is that *g*′ is derived for long Gaussian coils, which may not be applicable in the case of smaller branched macromolecules (Snejdrova et al., 2020).

Branched initiators must be well esterified; the degree of PLGA esterification is determined using ^13^C NMR, based on the peak shift of the branched initiator from 3.7 ppm (free) to 4.1 ppm (esterified). ^13^C NMR is used to assess samples for peaks indicative of ^13^C-labeled glucose and to determine the blockiness of the polymer based on the shift of the glycolide carbonyl group from 166.3 ppm (adjacent to another glycolide) to 166.4 ppm (adjacent to another lactide) (Hadar et al., 2020).

## 4. PLGA–drug interactions

The physicochemical properties of the incorporated drug can significantly impact the release rate profiles of PLGA microspheres (Han et al., 2016). Moreover, the interactions that occur between PLGA and active drugs may greatly affect the process parameters of microspheres (Jain, 2000).

### 4.1. Interactions with small molecule drugs

Polymer–drug interactions have been reported to play a critical role in controlling drug release characteristics. If the drugs encapsulated in microspheres are weak bases or acids, the drug-induced polymer degradation should be evaluated (Jain, 2000). Basic drugs can catalyze the hydrolysis of ester bonds, accelerating polymer degradation and drug release. Meanwhile, basic drugs may also shield the terminal carboxyl residues, thus interfering with the autocatalytic effect and slowing down polymer degradation and water penetration into the matrix (Miyajima et al., 1998, 1999). Siegel et al. investigated how the release of PLGA is affected by drug loading and found that even if the rate of hydrolysis is accelerated by the carboxyl terminal groups, the intensity of interaction between the drug and PLGA may result in higher drug loading and a slower rate of release (Siegel et al., 2006). Higher drug loading means that a greater proportion of drugs will be encapsulated in the microspheres, and their effect on the polymer will be more prominent. Similarly, D’Souza et al. (2015) studied the effect of weakly basic nucleophilic drugs (risperidone and olanzapine) on the degradation of PLGA in microspheres and found that the Mw of PLGA decreased significantly when risperidone or olanzapine were loaded into the microspheres with a good correlation detected between decreased PLGA Mw and the quantity of drug loaded into the microspheres. The authors, therefore, postulated that weakly basic nucleophilic drugs may accelerate the degradation of PLGA. In this context, a nucleophilic compound refers to a molecule that promotes ester hydrolysis via nucleophilic catalysis, as seen in polymer scission, which occurs during the degradation of lactide and glycolide-containing biodegradable polymers. Such compounds are more effective nucleophiles toward ester groups of the polymer than are hydroxide ions or water. These compounds could include amines, carboxylate anions, active agents (such as risperidone, naltrexone, and oxybutynin), or inactive agents (such as choline, ethanolamine, and tri-ethanolamine) (Wright et al., 2003). Kumar & Palmieri (2010) found that the alkaline effect and the catalytic effect of the tertiary amine groups of risperidone become more pronounced as the quantity of the drug loaded into the microspheres increases. In this case, higher Mw (viscosity) PLGA is required to ensure sustained drug release from the microspheres.

Drug loading can also alter microsphere morphology. Bragagni et al. (2018) found that drug-free microparticles exhibit a regular spherical shape with a smooth surface, no visible pores or cavities, and a relatively homogeneous distribution of polymer. Interestingly, prilocaine-loaded microparticles were found to be spherical, however, had large pits on the surfaces. Therefore, the chemical nature and quantity of the drug loaded into a PLGA microsphere can influence several properties of the microsphere.

### 4.2. Interactions with peptide drugs

Currently, most of the sustained-release PLGA microspheres approved by FDA are loaded with peptide drugs. However, the chemical interactions between PLGA and peptides pose a significant obstacle for the successful development of these formulations. The nucleophilic primary amine groups (such as N-terminal and lysine side chains), and the carboxylic acid end groups of PLGA or PLGA degradation products can interact to form acylated adducts, which may have harmful effects, including loss of activity, immunogenicity, and toxicity (Houchin et al., 2007; Houchin & Topp, 2008). Generally, the electrostatically driven sorption of the peptide to PLGA is a common precursor to its acylation and is followed by the release of the acylated peptide. Many studies have focused on inhibiting the acylation of polypeptide drugs in PLGA. For instance, Zhang & Schwendeman (2012) found that peptide acylation is strongly inhibited in formulations containing divalent cations and/or carboxymethyl cassava starch as excipients. Moreover, Jiwei et al. (2019) neutralized the inner pH of microspheres, to varying degree, using Ca(OH)_2_; the polymer degradation rate, drug release rate, polymer degradation mechanism, and oligomer accumulation state within the microsphere are all affected. Houchin & Topp (2008) further summarized the chemical degradation reactions of peptide/protein in PLGA microspheres and their mechanisms, while discussing certain methods for stabilizing these drugs in PLGA systems.

## 5. Effect of microcosmic process parameters on macroscopic properties

**Figure 5.**
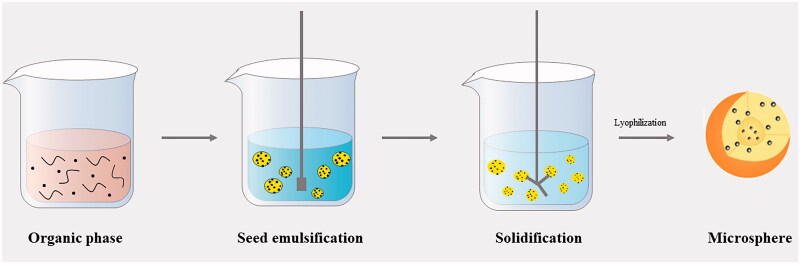
The preparation process of PLGA microspheres.

The study of PLGA microspheres can be split into the prescription and manufacturing process. Prescription factors include PLGA and an active drug, which have been discussed in sections ‘A comprehensive characterization of PLGA’ and ‘PLGA–drug interactions’, respectively. While there are several techniques for preparing PLGA microspheres, here, we will discuss the traditional emulsion-solvent evaporation method, commonly used in the manufacture of PLGA microspheres. The emulsion-solvent evaporation method involves the preparation of an oil-in-water emulsion. Specifically, a small quantity of non-polar organic solvent containing the polymer and drug (oil phase) is added to a polar solvent (water phase), containing a stabilizer (Figure 5). The organic solvent then evaporates or is extracted into the external aqueous phase (continuous phase). The dissolved PLGA molecules condense, resulting in the formation of shells. As the organic solvent continues to leave the system, the embryo microparticles contract and form a locally dense drug-PLGA microstructure in the whole microparticles. The PLGA microspheres are solidified by solvent removal (or solvent quenching), which is similar to the thermal quenching of amorphous polymers from the molten state (Bock et al., 2012). Water molecules also simultaneously diffuse into the microspheres and exchange with solvent molecules. After drying, the space occupied by water and residual solvents forms a void. Therefore, minor modifications in the manufacturing process, such as changes in the solvent–co-solvent system; ratio of continuous and dispersed phase; as well as polymer, drug, and surfactant concentrations, can influence the microspheres characteristics and the release profile of the drug (Rawat & Burgess, 2010).

### 5.1. The organic phase

As shown in Figure 5, the organic phase comprises the organic solvent (and co-solvents), PLGA, and the active pharmaceutical ingredient. Different solvents have different rates of solvent diffusion and evaporation, which reportedly impact the inner structure and porosity of microspheres (Xiao et al., 2013). The choice of solvent determines the distribution and solubility of the drug in the microspheres, particularly when the drug is hydrophobic, thereby affecting the drug loading and drug release kinetics. Residual solvent in PLGA microspheres also has a significant effect on the drug release kinetics: the miscibility of water and solvent affects the kinetics of solvent extraction, leading to differences in matrix density and porosity (Park et al., 2019). The rate of solvent evaporation directly influences the level of organic solvent in the hardening bath, which in turn can affect the chemical potential gradient of the species across the particle hardening surface, and consequently, the rate of solvent removal. Organic solvents, such as dichloromethane (DCM) and ethyl acetate (EA), are generally used. DCM is a class II organic solvent with a solubility of 2% v/v in water, and a boiling point of 40 °C, making it easy to remove. Shen et al. (2015) prepared risperidone microspheres using DCM and EA. Those made with DCM were smooth and spherical, with a less porous structure; while those made using EA had more irregular shapes and indentations, with a morphology similar to that of Risperdal^®^ Consta^®^. This may be attributed to the partial miscibility of EA and water (the solubility of EA in water is 8.7% w/w and that of water in EA is 3.3% w/w), resulting in dynamic movement of the two during the solidification process. This movement results in water inclusion in PLGA microspheres, generating irregular shapes and indentations during the drying process. However, DCM is a class II organic solvent, meaning that residual solvent levels are restricted to 0.06% in the Chinese Pharmacopoeia and 600 ppm in the United States Pharmacopoeia. Therefore, it is necessary to strictly control these levels. Alternatively, the high solubility of EA in water can cause excessively rapid solvent removal, leading to adhesion of the microspheres. This partial miscibility can also cause water inclusion during the microsphere solidification phase, leading to the formation of a highly porous core structure (Sah, 1997). To circumvent this issue, microspheres can be prepared via step-by-step solidification.

When high drug loading is required, it is necessary to add co-solvents to promote drug dissolution. For example, the drug loading in risperidone (Risperdal Consta^®^) and naltrexone microspheres (Vivitrol^®^) is 38% and 33%, respectively. In these cases, benzyl alcohol (BA) may be added to aid dissolution. To achieve this, the drug solution is uniformly mixed with the PLGA solution, evenly dispersing the drug molecules in the PLGA matrix, to facilitate their slow release from the tight matrix in the microsphere. Co-solvents have been reported to affect the rate of partitioning of the organic phase into the external aqueous phase and, therefore, influence the physicochemical properties and release kinetics of the drug (Rawat & Burgess, 2010). At times, drug loading in microspheres can decrease with increasing co-solvent concentration, which may result from changes in solubility of the drug in the organic solvent. Alternatively, it could also be related to the compatibility of organic solvents with water, which can lead to the diffusion of drugs into the external aqueous phase during emulsification, solvent evaporation, or extraction. For example, the solubility of EA in water leads to rapid PLGA precipitation and limits the movement of drugs on the surfaces or outer layers of the microspheres (Lu et al., 2014).

The concentration of PLGA in the organic phase can greatly affect the entrapment efficiency of microspheres. That is, a low concentration of PLGA produces an organic phase with low viscosity, allowing drugs (especially small molecule drugs) to escape into the aqueous phase and reducing the encapsulation efficiency of the microspheres. Contrarily, a high concentration of PLGA will produce a high viscosity of the organic phase, which resists the movement of drugs into the aqueous phase. Additionally, it would allow faster deposition of PLGA around the core material, resulting in a tighter microsphere matrix. During the process of drug release, a tighter microsphere matrix increases the time taken for the release medium to enter the microsphere, thus slowing the rate of PLGA degradation and prolonging the release time.

Moreover, drug concentration can be affected by the interaction between the drug and PLGA, as seen with weakly basic drugs that reduce the viscosity of the organic phase. This has been discussed in detail in Section 4.1 (Wright et al., 2003).

### 5.2. The seed emulsification phase

In the listed microspheres, the continuous phase used is generally an aqueous solution of polyvinyl alcohol (PVA). Here, the higher the PVA concentration in the aqueous solution, the stronger the emulsifying capacity, and the smaller the microspheres. Dawes et al. (2009) found that the drug loading of dexamethasone in larger microspheres (1%) was lower than in smaller microspheres (11%). This may be attributed to the difference in surface areas; small microspheres have a larger polymer–water interface, which enables them to encapsulate larger quantities of drugs. The rate at which water enters the polymer during the release phase increases as the surface area per unit volume increases, allowing dexamethasone to be released faster.

Additionally, the pH of the continuous phase can be adjusted to increase the encapsulation efficiency of small molecule drugs. If the continuous phase is made alkaline, weakly basic drugs exist in their less-soluble free base form, and the saturated concentration inhibits the escape of the drug into the aqueous medium. Alternatively, if the continuous phase is made acidic, weakly basic drugs would form salts with the acid, making them more soluble in the aqueous medium and reducing the encapsulation efficiency of the microspheres. It is worth noting that when the organic phase is mixed with the continuous phase, PLGA often precipitates at the interface, thus decreasing the encapsulation efficiency of the microspheres. A feasible solution to this is to add saturated organic solvent to the continuous phase to reduce the precipitation and loss of PLGA.

Figure 5 also depicts the seed emulsion stage of emulsification. Here, the drug and polymer are emulsified into micro-sized droplets. Standard processes using high-speed emulsifiers or ultrasound usually produce particles with a wide particle size distribution and varying internal and external shapes (Freitas et al., 2005) depending on the formulation and process parameters. New production processes involve membrane sieving and the use of microfluidic devices, allowing for the production of monodisperse microspheres, with a narrow size distribution (Samadi et al., 2013; Kazazi-Hyseni et al., 2014); however, there are few cases in which they have been successfully magnified and approved for marketing. Currently, high-pressure homogenization remains the primary method for the manufacture of the listed PLGA microspheres. High-pressure homogenization uses high-pressure strokes to drive the coarse pre-emulsion through interaction chambers composed of defined microchannels. Here, the product is accelerated to a high velocity and subjected to intense shear, impact, and cavitation. The size and distribution of the particles depend on the speed and duration of homogenization, as well as the width and depth of the container.

### 5.3. The solidification phases

The physical chemical events that take place between emulsification and microsphere hardening can be split into different stages: the diffusion of the organic solvent from the embryonic particles into the aqueous hardening bath, evaporation of the solvent, polymer phase separation at the microsphere surface, particle coalescence, and drug loss into the hardening bath. Two important concepts underlie the mass transport at the liquid–liquid interface, namely, turbulence and velocity distribution. The nature of turbulence induced by the impeller controls the size and speed of the smallest eddies that approach the surface of evaporation. The velocity distribution in the viscous sublayer is then affected by the eddies approaching the free air/water surface (Wang et al., 1999). In the aqueous medium, the organic solvent is exchanged with the aqueous solution resulting in PLGA precipitation in the spheres. The temperature of the solidification medium, pH, and pressure of the curing environment must be controlled during this phase.

The temperature affects the encapsulation efficiency of the microspheres. That is, drug solubility is higher when the temperature of the medium during the organic–aqueous exchange is elevated, leading to an increased amount of drug in solution. However, this allows the polymer chains to remain above the Tg for a longer duration, resulting in prolonged polymer molecule flexibility, which allows drug molecules to diffuse into the external phase. This results in a decrease in encapsulation efficiency. Many studies have reported the use of low temperature to reduce drug loss and achieve high encapsulation efficiency (Kang et al., 2014; Andhariya et al., 2019a). During isothermal solvent extraction, the motion of the PLGA chains is minimized at different rates, depending on where in the emulsion droplets they harden. Solvent molecules must diffuse over the radius of particles, thus, the distance they must travel to reach equilibrium is much larger than their respective sizes. Therefore, this requires a longer time period than temperature-induced glass transition. When the solvent in the PLGA microemulsion droplet is removed, the polymer with higher Mw, in the region of higher PLGA concentration, will begin to precipitate (i.e. become glassy), resulting in a high local chain density. The polymer forms a network of particles with uneven local density, which may be critical to the drug release characteristics of the microsphere. Solvent extraction also involves water uptake into the PLGA matrix. Water acts as a plasticizer, reducing the Tg. The moisture is then removed by vacuum drying or lyophilization (Bouissou et al., 2006; Park et al., 2021).

The reason for controlling the pH of the curing medium is the same as that for the continuous phase, which is detailed in section ‘The seed emulsification phase’.

The pressure of the solidification environment can also affect the rate of solvent removal and, therefore, the porosity and compactness of the microsphere matrix and drug distribution. Moreover, the invisible internal structure and drug distribution of microspheres make it difficult to identify the critical quality parameters. Under the conditions of normal temperature and pressure, the solvent evaporation/extraction is relatively slow, and the drugs in the emulsion may first crystallize out; continued evaporation would then lead to accumulation of crystals, which migrate outwards in the microsphere. At the end of the long-term solidification process, many drug crystals would have migrated out, distributed near the surface, and escaped from the microsphere. This would result in reduced encapsulation efficiency and would increase the risk of burst release. Under negative pressure (vacuum), the solvent volatilizes rapidly, and the PLGA in the emulsion rapidly precipitates into the spheres, reducing the risk of drug deposition and migration. Therefore, reduced pressure conditions result in higher encapsulation efficiency, decreased matrix porosity, and a uniform distribution of the drug in the microspheres. Andhariya, Janki et al. (2019) created risperidone microspheres of low porosity, with approximately 40% drug loading, using organic solvents in vacuum. The release properties were similar to those of the risperidone microsphere marketed formulation. Gu et al. (2015) transferred PLGA emulsion into an aqueous PVA solution (0.1% w/v), stirring at 600 rpm under vacuum, to form dexamethasone-loaded microspheres. The drug loading was found to be approximately 8% w/w, with a slight increase in the loading when the proportion of the more hydrophobic polymer, DLG7E, was increased.

The internal pore structure of PLGA microspheres plays a major role in the release characteristics of microspheres (Mao et al., 2007). Therefore, it is crucial to optimize the temperature, pressure, and pH conditions during the solidification phase to achieve optimal drug loading and release characteristics.

### 5.4. The drying phase

The drying process has also been shown to affect PLGA microsphere properties (Deshmukh et al., 2016; Wu et al., 2019). After solidification, PVA and other non-volatile organic solvents must be removed from the microspheres. Residual surface activity of the microspheres increases the plasticity of PLGA, in turn affecting the Tg and release characteristics of the polymer. Mass occupied by residual surfactants may cause the encapsulation efficiency of the microspheres to appear lower than it is. As most of the surfactants used are soluble in water, washing the microspheres with water generally removes the residual surfactants. The microspheres are then dried by freeze-drying, which introduces a certain degree of fluidity to them. After drying, pores form in the positions originally occupied by water molecules. These can be observed by profile analysis of the microsphere. If the microspheres have a high residual water content, their hydrolysis is often accelerated. Therefore, efficient drying of microspheres is essential.

The final stage of the drying phase may include an annealing process. In this phase, the drying temperature must be maintained at, or as close to, the Tg of the polymer as possible.

## 6. Continuous manufacturing process

PLGA microspheres are complex formulations, thus, minor changes in their manufacturing process can significantly affect drug release characteristics. One of the major challenges in clinical and commercial development of sub-micron polymeric particle formulations is scaling up their production without affecting the formulation specifications defined at the lab scale. To avoid inconsistencies in the formulation, currently listed microspheres adopt the method of continuous production. Since the control of process parameters is crucial in the manufacture of microspheres, alternative production techniques with ‘seamless’ scalability should be explored. In this respect, continuous processes are beneficial, as they allow the termination of production when the target quantities have been produced, without altering the process parameters based on the production scale (Desai, 2012; Ranjan et al., 2012; Ye & Squillante, 2013; Paliwal et al., 2014).

To date, the apparatus and methodology for preparing microparticles, using in-line solvent extraction, have been developed. First, the organic phase and continuous phase are mixed, adjusting the flow rate to transfer them to a static mixer for emulsification using high-speed homogenization. The emulsion is then combined with an extraction liquid in a blending static mixer. The outflow of the blending static mixer is mixed with additional extraction liquid and the emulsion is transported to another mixed static mixer containing the extract and subsequently combined with other extracts.

**Figure 6.**
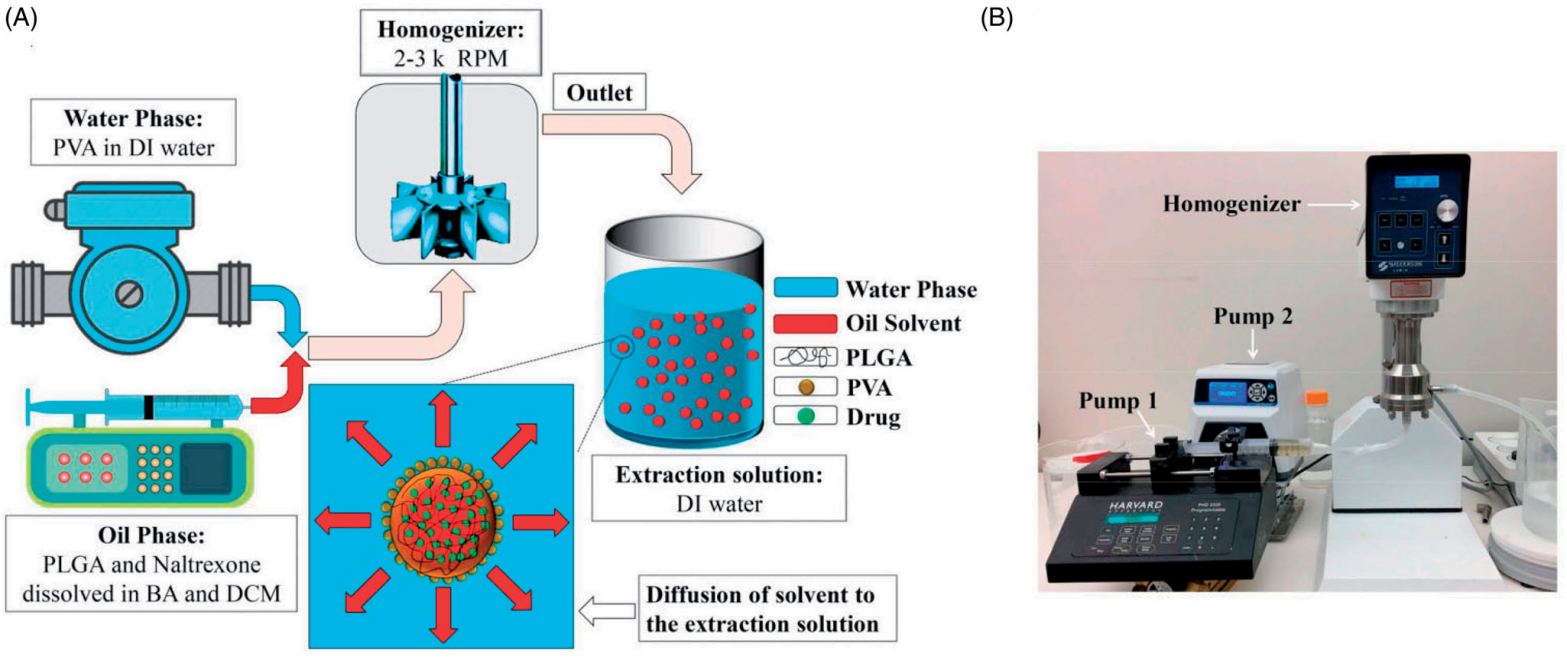
(A) Schematic of naltrexone-loaded PLGA generation using an in-line emulsification-extraction process. (B) Experimental set-up used to generate particles by pumping both oil- and aqueous-phases into the homogenizer and transferring the emulsion to the extraction solution (Sharifi et al., 2020).

Although considerable manpower, as well as materials and financial resources, are invested in the study of PLGA microspheres, it remains impossible to replicate industrial scale production. Laboratory studies show that solution mixing, homogenization, and solidification are not continuous, and significant differences are observed between the batches of microspheres, making it difficult to standardize these parameters during industrial production. Sharifi et al. (2020) studied a naltrexone-loaded PLGA microsphere formulation, made using an in-line homogenizer, with a flow rate of 100 mL/min for the oil phase. This continuous production approach produced PLGA microspheres with reproducible size distribution, drug loading, and drug release rate (Figure 6). Meanwhile, Operti et al. (Operti et al., 2018) explored three well-established process technologies for continuous large-scale production of sub-micron PLGA particles, developed on a lab scale, using batch production. These studies can realize the on-line preparation of microspheres in laboratory research, which may serve as a bridge for the development of process parameters required for scale-up production.

## 7. Critical quality attributes of PLGA microspheres

The CQAs of PLGA microspheres (drug loading, particle size, morphology, and porosity) are sensitive to minor changes in the manufacturing processes and can affect drug release characteristics and product performance. Biopharmaceutical manufacturing processes that were developed before the implementation of QbD typically use control strategies that are not developed based on a formal understanding of criticality. Therefore, it is important to understand the CPPs and CQAs of the product (Mollah et al., 2013). *In vitro* drug release profiles and the CQA of the microspheres are affected by the physicochemical properties of the polymer (Mw, crystallinity, monomer ratio, and monomer sequence) and the encapsulated drug. The CQAs of PLGA microspheres are discussed in this section.

### 7.1. Drug loading

Drug loading and encapsulation efficiency are important indicators of drug content in microspheres (Di et al., 2020). Drug loading not only affects the drug release characteristics, but is also reflective of the inter-batch stability, which is an important indicator of process maturity. Drug loading is determined by dissolving a known quantity of the microspheres in a suitable solvent, such as dimethyl sulfoxide, methanol, or ethanol, based on the solubility of the matrix and active components, to release the free drug. The concentration of drug in solution is generally determined by high-performance liquid chromatography (HPLC). Drug loading is calculated according to the following equation:

(2) $$\text{Drug loading}=\frac{\text{weight of drug entrapped}}{\text{weight of microspheres}}\times 100$$

### 7.2. Particle size and particle size distribution

Particle size affects the needle penetration and drug distribution (Di et al., 2020). Moreover, a linear relationship exists between the particle size and degradation rate. In smaller particles, the degradation products can readily diffuse to the surface, whereas in larger particles they must travel a longer distance to reach the surface, during which time, autocatalytic degradation of the remaining polymer can occur (Dunne et al., 2000).

Sieving method, light microscopy (LM), light resistance method, and laser light scattering (LLS) can be used for particle size evaluation (Alagusundaram et al., 2009; Burgess et al., 2004). LM is used to investigate the microsphere coating parameter by visualizing microspheres before and after the coating process. However, the disadvantages of this method include low resolution and the requirement for large sample sizes to obtain reliable results. Currently, LLS is the most widely used method for particle size analysis of various dosage forms. This technique functions on the basis that particles of different sizes scatter light in different directions. Therefore, detectors can be placed at different angles around the sample to measure changes in the light energy distribution, allowing particle size to be calculated. Particle size distribution can then be expressed either as a particle size distribution diagram or as the polydispersity index (PDI) and span. A small span and PDI indicate uniformity in particle size.

(3) PDI=SD/d

where SD represents the standard deviation of particle size and *d* is the average particle size. PDI is determined using the laser diffraction particle sizer.

(4) $$\mathrm{Span}=\left(D_{90}-D_{10}\right)/D_{50}$$

where *D*_90_, *D*_50_, and *D*_10_ represent the particle size corresponding to the cumulative frequency of 90%, 50%, and 10%, respectively, in the particle size distribution map.

### 7.3. Morphology

Optical LM and scanning electron microscopy (SEM) are the most widely used methods to obtain detailed information on the surface morphology of microspheres. SEM is primarily used to analyze their shape and surface, as well as the cross section of microspheres to reveal their internal structure. SEM uses electrons to produce high resolution (10–20 nm), topographic, three-dimensional images, whereas LM produces two-dimensional images of a lower resolution (200–300 nm) (Prajapati et al., 2008).

### 7.4. Porosity

Microspheres with a high porosity allow water to enter easily and access the ester linkages of the polymer. This facilitates the escape of the drug from the microspheres, without a prolonged lag phase. Alternatively, less porous PLGA microspheres have a longer lag phase due to the reduced water accessibility (Mollah et al., 2013). As pore size and pore distribution are difficult to control (Liao et al., 2016), the use of rapidly degrading microspheres has been explored. Hence, it is necessary to focus studies on the porosity of microspheres to characterize microsphere degradation.

A mercury intrusion porosimeter is often used to determine the percent porosity and the average pore diameter of microspheres. The microspheres are tested at a mercury filling pressure of 0.53 psi, and the total percent porosity, average pore diameter, total intrusion volume, and total pore area are recorded.

(5) $$\mathrm{Porosity}(\%)=(1-\frac{\text{bulk density}}{\mathrm{apparent}(\mathrm{skeletal})\mathrm{density}})\times 100$$

### 7.5. Transition glass temperature (Tg)

The Tg of PLGA is an important parameter related to its structure and properties and depends on the free volume of the polymer (Allison, 2008). The Tg value corresponds to the interactions between the chains and the Mw. Higher Mw polymers have a smaller free volume at the end of the chain and, thus, have a higher Tg energy. Therefore, Tg increases with Mw. The *B* value, which quantifies the degree of branching in a non-linear polymer, of the linear reference is 2. That is, the higher the *B* value, the higher the degree of branching of the sample. For the microsphere preparation of non-linear carrier, the smaller the value of *g*′, the higher the value of *B*, and the lower the free volume will be (Snejdrova et al. 2016).

The Tg of PLGA is affected by the physical aging of microspheres at high storage temperatures. The free volume of the polymer decreases during physical aging, which results in an increase in its density (White, 2006), thus, causing an increase in the Tg of the microspheres. This increase in Tg alters the glass transition state of the polymer from amorphous to crystalline, causing physical deformation of the microspheres and changes in their release characteristics.

The Tg of naltrexone microspheres is often analyzed using a modulated temperature differential scanning calorimeter. Experiments have been performed in hermetically sealed pans, using a heating rate and a modulation amplitude with small temperature differences, with a modulation period. A few milligrams of the microspheres are required for this method, and the Tg is determined as the glass transition midpoint in the reversing signal (Andhariya et al., 2019b).

### 7.6. Water contact angle

The contact angle is used to analyze the surface wettability and hydrophilicity of polymers. Contact angles <90° are indicative of high wettability, and favor the diffusion of liquid on the materials. Meanwhile, contact angles >90° indicate low wettability (Zhu et al., 2019). Wang et al. (2015) studied the relationship between microparticle hydrophobicity and adjuvanticity by preparing polylactic acid (PLA-, PLGA-, and PELA-based microsphere particles via premix membrane emulsification). The resulting particles were similar in size and morphology, however, had different surface hydrophobicity. The contact angles of PLA, PLGA, and block copolymers formed by polyethylene glycol and poly(l-lactic acid) (PELA) were 90°, 76°, and 58°, respectively, which reflects the difference in their hydrophobicity. That is, PLA was the most hydrophobic, whereas PELA was the least.

The sessile drop method is often used for the measurement of static water contact angles. Briefly, a water droplet is placed on the dry surface of each composite, and the angle of contact between the water and composite is detected at room temperature using a contact angle meter, equipped with a special optical system and a charge coupled device camera.

## 8. Conclusions and future prospects

The complexity and poor reproducibility of PLGA microspheres in large-scale manufacturing are major obstacles to their development as drug delivery systems. This review has evaluated the composition of PLGA microspheres and the technology involved in their production, based on the QbD concept, as a thorough understanding of PLGA is essential for the development of microspheres. Further investigation into the interaction between PLGA and different drugs is required to avoid the risks associated with development of microspheres. From understanding the process of PLGA microsphere manufacture, we know that small modifications in the process parameters can cause significant changes in the quality properties of the microspheres. To overcome the challenge of poor process reproducibility, we propose the application of continuous manufacturing processes for such formulations. Finally, we presented potential CQAs of the microspheres that can be used as a basis for optimizing process parameters.

Recently, there have been numerous new methods reported for the preparation of PLGA microspheres. For protein-loaded microspheres, active self-encapsulation (ASE) is a post-loading method based on absorption of positively charged proteins in microporous PLGA microspheres loaded with negatively charged polysaccharides (trapping agents) (Scheiner et al., 2021). Furthermore, microfluidic systems represent a platform for the production of monodisperse microspheres as they can fabricate PLGA microspheres in a controlled and reproducible manner using the oil/water microemulsion method in microfluidic channels, achieving a uniform sustained-release profile of drugs from the microspheres. Additionally, CFD modeling is used to investigate droplet flow in the microfluidic channel and to simulate the preparation of PLGA microsphere in the microfluidic chip (Jafarifar et al., 2017; Chengcheng et al., 2019). Furthermore, Shirasu porous glass (SPG) premix membrane emulsification has been employed to ensure controlled particle size as the SPG membrane is a porous glass membrane; the dispersed phase passes through the pores of the microporous membrane to form droplets on the surface of the membrane with the action of nitrogen pressure. Under the flushing action of the continuous phase flowing along the membrane surface, the diameter of the droplet reaches a certain value and will be peeled off from the membrane surface to form an emulsion. The microporous membrane with uniform pore size can then be used to control the particle size and distribution of the emulsion (Feng et al., 2014). However, these methods remain far from industrialization.

Collectively, the work presented in this review demonstrates that PLGA is a biodegradable, safe, and reliable polymer that can be used for drug delivery. In fact, more than 10 PLGA microsphere formulations have been approved by the FDA. Accordingly, research focused on PLGA-encapsulated vaccines has become a hot topic, especially given the need for safe and effective vaccines during the ongoing SARS CoV-2 pandemic. Hence, accelerating the research on PLGA microspheres, and other PLGA preparations, serves to benefit people's livelihood and protect it from diseases through the development of effective drug delivery systems.
